# Development and internal validation of prognostic models for event-free survival in newly diagnosed Ewing sarcoma patients based on routinely collected clinical characteristics from the European randomised controlled trial, EE2012

**DOI:** 10.1136/bmjopen-2023-082941

**Published:** 2024-12-20

**Authors:** Laura Kirton, Richard Riley, Piers Gaunt, Bernadette Brennan, Kym Snell

**Affiliations:** 1 Cancer Research UK Clinical Trials Unit, School of Medical Sciences, College of Medicine and Health, University of Birmingham, Birmingham, UK; 2 Department of Applied Health Sciences, School of Health Sciences, College of Medicine and Health, University of Birmingham, Birmingham, UK; 3 NIHR Birmingham Biomedical Research Centre, Birmingham, England, UK; 4 Royal Manchester Children's Hospital, Manchester, UK

**Keywords:** Prognosis, Sarcoma, Paediatric oncology, STATISTICS & RESEARCH METHODS

## Abstract

**Abstract:**

**Introduction:**

Ewing sarcoma is a rare paediatric cancer. Currently, there is no way of accurately predicting these patients’ survival at diagnosis. Disease type (ie, localised disease, lung/pleuropulmonary metastases and other metastases) is used to guide treatment decisions, with metastatic patients generally having worse outcomes than localised disease patients. However, not all patients fit this trend. An accurate prognostic model could be used to guide treatment decisions in clinical practice to avoid patients being incorrectly under or overtreated.

**Methods and analysis:**

This study aims to develop and internally validate prognostic models in newly diagnosed Ewing sarcoma patients, using the EE2012 clinical trial data set. The models will incorporate prognostic factors, identified from a literature review, to predict patients’ probability of event-free survival at clinically important time points. Three models will be developed, for comparison of their performance and stability, using different methods of model selection and penalisation for overfitting (full model or backwards selection applying uniform shrinkage; and lasso variable selection). The models will be internally validated using bootstrapping to give optimism-adjusted performance statistics (calibration and discrimination) and model stability results. Patient and clinical user groups will be asked to determine risk thresholds to guide treatment decisions in clinical practice based on the model. Decision curve analyses will examine clinical utility at these thresholds.

**Ethics and dissemination:**

This study is a secondary analysis of EE2012 clinical trial data. The EE2012 trial received ethical approval from the competent authorities (UK ethics reference approval number 12/NW/0827). This study is covered by the trial ethics in place. The results from this study will be published in peer-reviewed journals to act as a benchmark for future studies.

**Trial registration number:**

EudraCT number 2012-002107-17. ISRCTN number 92192408.

**STRENGTHS AND LIMITATIONS OF THIS STUDY**

- This study considers multiple model selection approaches and methods for dealing with overfitting.
- The set of candidate predictors are limited to variables routinely collected at diagnosis in the EE2012 trial, and therefore potentially important variables (eg, race) cannot be included in the model.
- This study will use good quality data from a prospective randomised controlled trial. However, given that EE2012 is reaching trial closure, any remaining issues with the sample size, functional form of variables and generalisability that could influence this study can no longer be addressed.
- Robust bootstrapping methods will be utilised to quantify the models’ stability and perform internal validation to produce optimism-adjusted results.
- The decision curve analysis will examine clinical utility of the models, with suitable ranges of risk thresholds being identified prior from patients and clinicians.

## Introduction

 Ewing sarcoma is a bone and soft tissue cancer, predominantly occurring in adolescents and young adults.^1^ It is a rare disease, with less than 70 diagnoses per year in the UK.^2^ Patients diagnosed with Ewing sarcoma have varying outcome with little understanding as to how patient characteristics drive response to treatment. There are several known baseline prognostic factors; however, they have not yet been combined to give a usable prognostic tool with event-free survival (EFS) as the outcome.

Other groups have attempted to develop prognostic models/risk stratification tools in newly diagnosed Ewing sarcoma.^3^,^6^ There are key limitations to these studies, for example, issues with sample size, not adjusting for overfitting, not assessing calibration, not addressing the clinical utility of the model, etc. Therefore, developing an original prognostic model, while considering the robust statistical approaches required for a reliable model with precise and accurate predictions, means it can act as a benchmark for subsequent studies (eg, biomarker studies).^7^

Patients are currently only stratified in clinical practice based on their disease type (ie, localised disease, lung/pleuropulmonary metastases or other metastases). Metastatic patients are generally worse performing than localised disease patients and are more likely to be offered experimental treatments in clinical trials.^8^ However, there are discrepancies within each of the three groups of disease type.^9^ Localised disease patients are split into high and low risk groups of progression, and this is only identified partway through treatment at the point of local therapy.^10^ This time point is considered too late as this high-risk group generally do as poorly as the metastatic patients and may benefit from more intensive treatment following diagnosis. Alternatively, some patients with metastases do well even with the standard treatment; if patients present with metastases at diagnosis and are subsequently offered experimental treatment, there may be potential for unnecessary additional treatment burden. A prognostic tool at diagnosis would enable this risk to be predicted much sooner for patients to receive a more tailored treatment plan.

Stratifying patients according to their probability of survival would enable more personalised treatment, and subsequently improve survival in each subgroup of patients. Due to the age group of these patients, it is equally important not to overtreat patients as it is to undertreat them. Thus, tailoring treatment to patients according to their baseline characteristics would prevent the risks associated with over and undertreating.

### Objectives

This project aims to develop and internally validate a prognostic model that predicts patients’ risk of progression, recurrence, second malignancy, or death in newly diagnosed Ewing sarcoma, using data from the EE2012 clinical trial.^10^

A secondary objective is to compare three methods of model development: the full model (no variable selection) and backwards elimination with uniform shrinkage, and least absolute shrinkage and selection operator (lasso) variable selection. Each model will be internally validated, and their optimism-adjusted predictive performance and stability estimated and compared.

## Methods and analysis

### Study design

This protocol describes the secondary analysis of prospectively collected data from the EE2012 clinical trial, comparing two standard of care frontline chemotherapy regimens for the treatment of newly diagnosed Ewing sarcoma patients. EE2012 is a large, phase III, European, randomised controlled trial. The trial was conducted with full ethics approval and in accordance with Good clinical practice (GCP) standards, funded by Cancer Research UK (CRUK). The full details of the trial protocol can be found online.^10^

In summary, patients were randomised 1:1 between the two frontline chemotherapy regimens, making up their induction and consolidation treatment. Stratification variables were used in a minimisation algorithm for the randomisation to balance patient characteristics between the two treatment arms, VIDE and VDC/IE. The stratification variables were disease type, tumour volume, age, sex and country. The latter was a stratification variable to account for the differing strategies to recruiting patients with ‘other metastases’ between countries.

All other treatments patients received while on the trial were variable, including radiotherapy, surgery and high-dose chemotherapy. In addition, there was a second randomisation for the addition of Zoledronic acid to the consolidation chemotherapy. The first randomisation was a stratification variable for the second randomisation, meaning that the effects of Zoledronic acid were balanced across the treatment arms.

The primary outcome of this trial was EFS, where an event was defined as the time to the first progression, recurrence, second malignancy or death by any cause. Patients who did not experience an event were censored at their date last seen. Secondary outcome included overall survival and toxicity.

The trial had a positive result, finding the frontline chemotherapy regimen used in the USA (VDC/IE) had better survival than the regimen used across Europe (VIDE), with similar toxicity in each.^2^ As a result of this trial, the frontline chemotherapy regimen used for primary Ewing sarcoma patients is now VDC/IE.

The start date for this secondary analysis was August 2023, with the development of this protocol. The results of the secondary analyses are planned to be completed at the start of 2025, with the results being disseminated in the second/third quarter of 2025.

### Data source

The trial sponsor at the University of Birmingham owns all data collected from the EE2012 clinical trial. All necessary parties, including the Chief Investigator and Co-Investigator, Bernadette Brennan and Keith Wheatley, respectively, have granted full access to the data.

### Participants

Over the 5 years EE2012 was open to recruitment (December 2013 to May 2019), 640 patients were recruited from 10 countries, as described in the first results paper.^2^ All patients were followed-up for a minimum of 2 years, unless they died, withdrew or were lost to follow-up.

The inclusion criteria included any histologically and genetically confirmed Ewing sarcoma family of tumours of bone or soft tissue, or ‘Ewing’s-like’ but negative for EWSR1 gene rearrangement, age >2 and <50 years, randomised ≤45 days after diagnosis and no prior treatment other than surgery. Exclusion criteria included contraindication to trial treatment and second malignancies.

### Outcomes

The primary outcome for prediction is EFS, as defined above. The time points of interest for prediction are 2, 3 and 5 years, with 3 years being the primary time point of interest.

### Sample size

The sample size for this study is fixed at 640 participants, as this was the number of patients recruited to EE2012. The maximum number of candidate predictors for the model can be obtained through rearranging the proposed sample size formulae for developing a prognostic model,^11^ given a particular event rate, time horizon and expected performance. The key time point for prediction is 3 years, for which the event rate from EE2012 was 36%, with a mean follow-up of approximately 3.2 years. A previous prognostic model developed specifically for Ewing sarcoma patients to predict OS, reported a C statistic of 0.7.^3^ This corresponds to an adjusted Cox-Snell R^2^ of 0.146, which can be used as the expected explained model variance in the sample size calculation. Finally, a postestimation global shrinkage factor of 0.9 is targeted, to reflect 10% overfitting.^11^ Based on this information and use of the Riley *et al* approach, utilising the ‘pmsampsize’ command in Stata,^12^ up to 11 predictor parameters can be considered for model selection, to target small overfitting and precise estimation of the overall risk.

### Candidate predictors

Several potential predictors of EFS in Ewing sarcoma have previously been identified in systematic reviews.^13 14^ Similarly, previous prognostic modelling studies in newly diagnosed Ewing sarcoma have been conducted.^3^,^6^ From those, the potential predictors that may have prognostic ability and are measured at diagnosis includes metastatic site, number of metastases, tumour size, tumour volume, location of primary tumour, age, serum lactate dehydrogenase levels, sex and race. As part of the proposed research, these potential predictors will be limited to a set of 11 candidate predictor parameters. It is important that the predictors considered are endorsed by clinical experts in Ewing sarcoma. In addition, only variables that were routinely collected at diagnosis as part of the EE2012 trial will be included in the set of candidate predictors; these include randomised treatment, metastatic site, number of metastases, tumour volume, location of primary tumour, age and sex. An additional variable under consideration for inclusion in the set of candidate predictor parameters is whether the diagnosis was ‘Ewing’s like’ or not. Moreover, the set of candidate predictors will be chosen based on clinician and patient opinion, within the limitations of the EE2012 data set.

### Missing data

Missing data will be reported for each of the candidate predictors. As less than 1% of the EE2012 data is anticipated to be missing due to the routine collection of baseline variables, a complete-case analysis will be used for prognostic model development. If needed, a sensitivity analysis using the multiple imputation by chained equations method will be used to impute the missing values of the variables in the model.^15^

## Statistical analysis plan

### Exploratory analysis

Descriptive statistics will be used to summarise the candidate predictors. Binary and categorical variables will be summarised as frequencies with proportions (%) and continuous variables will be summarised as means with SD or medians with IQR, depending on their distributions. Additional exploratory analysis will include looking at correlations between candidate predictors.

The unadjusted prognostic effect of each candidate predictor with EFS will be summarised. These results will not contribute to model development or variable selection but will aid descriptive summaries of the data.

### Model selection

Multivariable Cox proportional hazards models will be developed using three model selection methods, with EFS as the outcome. Three models are used to compare their performance and stability, to motivate further external validation studies. All model coefficients will be reported as hazard ratios (HRs), with the baseline survival derived using a non-parametric estimate at each of 2, 3 and 5 years separately.

The three models will be generated using (1) a full model (ie, one containing all candidate predictors) followed by uniform shrinkage; (2) backwards elimination followed by uniform shrinkage and (3) Cox model with lasso penalty for variable selection, as now described in detail.

### Full model

All candidate predictors will be included in the model, regardless of statistical significance. Non-linearity of continuous variables will be examined using fractional polynomials (FPs). Maximum likelihood estimation will be used to fit the model. Uniform shrinkage will be applied to the predictor effects by multiplying the log HR estimates (betas) by a postestimation global shrinkage factor. This will be derived using the approach of Van Houwelingen and Le Cessie.^16^ For comparison, bootstrapping will also be used to derive the shrinkage factor, as detailed in the Internal Validation section below. This model will act as a comparator for the other two models.

### Backwards elimination

The model developed using backwards elimination allows a subset of predictors to be retained from the full set of candidate predictors. The approach begins with the full model, and whichever variable has the highest p-value over some prespecified threshold is removed. This process is then repeated with the remaining variables in the model, and iterated until all variables in the model are significant, using a p-value of less than 0.157,^17^ based on Akaike Information Criteria. Treatment, disease type (localised disease vs lung/pleuropulmonary metastases and localised disease vs other metastases), age, sex and tumour volume will be forced into the model as these are known prognostic factors in newly diagnosed Ewing sarcoma. Non-linearity of continuous variables will be assessed using the multivariable FP process, an additional step in the backwards elimination algorithm.^18^ FPs will be considered up to the second degree to allow for flexibility without the model becoming too complex, and the terms must significantly improve the model fit (p-value≤0.05) for them to be included in the model. Finally, a postestimation global shrinkage factor will again be applied to the covariates in the model to adjust for overfitting. This will be derived using bootstrapping for internal validation, as described in detail below, utilising the backwards elimination method when deriving each bootstrap model.

### Lasso variable selection

A common issue with the backwards elimination algorithm is the potential for omission of important predictors and inclusion of unimportant predictors, by chance, leading to overfitting and model instability.^19^ A potentially better approach is the lasso, which combines variable selection with shrinkage through the penalty terms added to the likelihood.^20^ That is, the approach can shrink the coefficients towards zero during the model estimation by penalising the likelihood that is being maximised; greater model variance and predictor effect uncertainty leads to greater penalisation. Shrinkage is applied during the variable selection process and will vary depending on the coefficient being estimated. A coefficient shrunken to zero is equivalent to the removal of that variable from the model, that is, variable selection. All variables in the model will be penalised and subsequently shrunken. Similar to before, non-linear terms will be explored using FPs to the second degree. A frequentist approach will be used to fit the Cox model with lasso penalty, but an equivalent Bayesian approach (where the lasso penalty is introduced via a Laplace prior distribution) will also be considered for comparison.

### Comparison of model stability

The model stability from each method of variable selection will be assessed using 500 bootstraps (per model), for each of the time points of interest, and compared.^21 22^ A more stable model is preferred as it is likely to be more reliable in new data.

### Model performance

Each of the models will be assessed at the key time points for their performance using discrimination and calibration statistics.^23^ Harrell’s C-index and a time-dependent C-index will be used as the measure of discrimination.^24^ Similarly, calibration plots including smooth calibration curves will be used to assess the models’ prediction accuracy at each of the relevant time points,^25^ derived using pseudo-observations.^26^

### Internal validation

The 500 bootstrap samples obtained to assess the model stability will also be used to obtain optimism-adjusted estimates of discrimination and calibration. These are calculated by fitting the full model in each of the bootstrap samples and calculating the performance statistics from the bootstrap model in the original sample. The difference in performance (of bootstrap model in the bootstrap data vs the original data) will be averaged across each of the bootstraps to obtain the optimism-adjusted statistics. The optimism-adjusted calibration can also be used as a uniform shrinkage factor.

### Sensitivity and exploratory analyses

If the proportional hazards assumption does not hold, then time-dependent covariates will be added to the model.^27^ Additionally, in the instance where the continuous variables in the models lack stability, an additional analysis will be performed using splines (with prespecified knot locations) instead of FPs.^28^ Finally, the deriving (or recalibrating) models directly using pseudo-observations themselves, at each time point separately, and comparing their stability to other models may be considered.^26^

### Decision curve analysis

Discussions with clinicians and patient advocates will provide an appropriate range of risk thresholds for the decision curve analysis.^29^ Since patients’ disease type (localised, lung/pleuropulmonary metastases and other metastases) is considered highly prognostic and is already used in clinical practice, a similar risk threshold may be considered.

To examine the potential clinical utility of the models, the net benefit (a function of sensitivity, specificity and outcome incidence) for each model will be calculated and plotted over a range of thresholds deemed clinically relevant for decision making, producing a decision curve. The decision curves for the different models will be compared, where the net benefit across thresholds will be compared with the net benefit of ‘treat all’ and ‘treat none’ strategies.^30^ Again, bootstrapping will be used to examine and adjust for optimism in net benefit.

### Statistical software

All statistical analysis will be performed using Stata V.18.0.

### Reporting

The project will be reported in accordance with the TRIPOD+AI guideline.^31^ In addition, the advantages and disadvantages of this study will be discussed in the context of the PROBAST tool.^32^

### Patient and public involvement

A patient and public involvement (PPI) working group has been formed to support this work for the duration of LK’s research project, including this secondary analysis: the development and internal validation of prognostic models in newly diagnosed Ewing sarcoma. The working group is composed of Ewing sarcoma and osteosarcoma patient advocates, and will meet regularly during the project. Indeed, the PPI working group has met numerous times to discuss this work (ie, prior to this study being funded), including gauging their perspectives on certain decisions required for this study (eg, choosing candidate predictors, deciding what threshold probabilities to use for the decision curve analysis, etc), discussing the potential impact of this study and beginning to think about how the findings could be implemented in clinical practice and communicated to the patients population.

## Discussion

There is clear clinical rationale for having a greater understanding of how newly diagnosed Ewing sarcoma patients are expected to perform at baseline beyond the disease type they present with. Developing and internally validating a prognostic model could give clinicians a tool to predict patients' risk of progression (and survival) and subsequently offer tailored treatment. The EE2012 trial is the perfect opportunity to use high-quality data to do this. Although, a limitation of this study is that the candidate predictors will be limited by the data collected in the EE2012 trial. Similarly, issues with sample size (ie, the number of candidate predictors that can be included during model selection), generalisability, etc, cannot be addressed for the purposes of this study as the EE2012 trial is reaching closure.

Considering the varying evidence to support a preferred model selection algorithm, using three different methods of model selection and assessing the stability of each is a comprehensive way of helping to expose and improve the robustness of models. The findings from these model comparisons may also support the wider methodological research question as to which method is most suitable, especially in the instance where the data set is smaller, and the population is rare.

Going forward, for any of the models to be used in clinical practice they should be externally validated and recalibrated if necessary.^33^ If a model demonstrates good predictive performance when validated, it could be implemented into the next large international randomised controlled trial in newly diagnosed Ewing sarcoma, INTER-EWING-1, to aid decisions around which randomisations patients could enter based on their risk prediction, for example, randomising the high-risk patients to standard of care versus standard of care with a novel therapy. In addition, the resulting models have the potential to be updated as additional prognostic factors derived from translational research (ie, biomarkers) arise, which may give more accurate prediction. Methodologically, an extension of methods put forward as part of the PROGRESS framework may be required to undertake this work in the setting of a rare disease.^7^

## Ethics and dissemination

This study is a secondary analysis of EE2012 clinical trial data. The EE2012 trial received ethics approval from the competent authorities (UK ethics reference approval number 12/NW/0827). The secondary analysis described in this protocol is covered by the trial’s ethical approval with agreement from the trial’s Chief Investigator, Bernadette Brennan, an author on this paper. Informed consent obtained for the EE2012 trial covered the use of patient data for future ethically approved research. The EE2012 trial’s Patient/Parent Information Sheet details that the trial data will be used to meet trial objectives and further research, for which the secondary analysis detailed in this protocol aims to do.

The results from this study will be published in peer-reviewed journals to act as a benchmark model for future studies.
