# Supplementary material for: Development and internal validation of prognostic models for event-free survival in newly diagnosed Ewing sarcoma patients based on routinely collected clinical characteristics from the European randomised controlled trial, EE2012
Source: BMJ Open. 2024 Dec 20;14(12):e082941. doi: 10.1136/bmjopen-2023-082941 (PMC11667367; doi:10.1136/bmjopen-2023-082941)
Supplement: Supplementary file 1 [file bmjopen-2023-082941.reviewer_comments.pdf]

PEER REVIEW HISTORY

BMJ Open publishes all reviews undertaken for accepted manuscripts. Reviewers are asked to complete a checklist review form and are provided with free text boxes to elaborate on their assessment. These free text comments are reproduced below.

ARTICLE DETAILS

Title (Provisional)

Development and internal validation of prognostic models for event-free survival in newly diagnosed Ewing sarcoma patients based on routinely collected clinical characteristics from the European randomised controlled trial, EE2012: Protocol

Authors

Kirton, Laura; Riley, Richard; Gaunt, Piers; Brennan, Bernadette; Snell, Kym

---

VERSION 1 - REVIEW

**Reviewer**	**1**
**Name**	**Mathew, Joseph**
**Affiliation**	**HealthCare Global Enterprises Ltd**
**Date**	**18-Feb-2024**
**COI**	**None**

The study describes the protocol that would be followed in the development and internal validation of a prognostic prediction model to predict EFS in patients with Ewing sarcoma (ES) at presentation by the retrospective evaluation of the EE2012 data (the prognostic variables having been identified from a review of literature).

The manuscript is well written and once validated, the model would serve as a valuable resource for early prognostication in ES. I have the following comments to make:

1. The protocol for the EE2012 has been described in brief which is good. Please also consider mentioning the geographical distribution of patients included in the study since the same will have a bearing on generalizability and replicability of the model.

2. With regard to the inclusion criteria, I assume that all patients with histology-proven ES and confirmed by molecular studies, regardless of organ of origin (skeletal vs extraskeletal) or metastatic status would be selected?

3. Please explain the rationale for including Ewing's like round cell neoplasms with conventional ESFTs in a prognostic model since it is now known that the biology and hence

the presentation, response to therapy and aggressiveness of these tumors differ from that of classic ES.

4. In the inclusion criteria, patients with "no prior treatment other than surgery" will be selected. Please clarify if this is a diagnostic biopsy that the authors are referring to or a surgical resection. In case of the latter, the surgical margin would represent an important prognosticator which cannot be overlooked.

5. Although all predictor variables included in the model are routinely measured in practice and hence has functional value, a significant limitation of protocol studies deriving data from RCTs is that the data may not include newer, more potent prognosticators. Moreover, despite EFS being a composite survival end point, it is influenced by a number of modifiable treatment-related factors including systemic therapy and regimen, radiological and/or histological response, local treatment and margin status (if surgically addressed), all of which may not be accounted for if only baseline variables are considered to generate a prognostic model for EFS.

6. Despite being a protocol study, I would expect a more detailed description of the prognosticators that would be included in the model, along with the rationale for selecting these variables and excluding others.

7. Also, I'm uncertain as to why the authors have included both tumor size and volume as candidate predictors, since the latter may be considered a function of size (unless indicative of overall tumor burden) and hence a more comprehensive parameter when compared to the largest dimension of a tumor.

8. Also, the EE2012 being a European trial, how "variable" a candidate predictor would race be?

9. How would the authors assess for risk of bias?

Minor comments and corrections:

1. The title of the study reads as "Developing and internally validating a

prediction model...". For clarity, I would suggest modifying this to a "

prediction model" as clinical prediction models could either imply diagnostic or prognostic prediction models.

2. Kindly recheck the references especially the sources that have been quoted from the net.

Comments to the Editor:

The article describes the protocol of a study which would retrospectively use prospectively-collected data to generate a prognostic model. Despite the drawbacks associated with the use of RCT data in developing clinical prediction models and prognostic marker validation, the approach may be considered acceptable as the authors have clarified the completeness of the EE2012 data, proposing measures to handle missing data if applicable.

The methodology followed in the study is standard. Nonetheless, being a protocol paper for the development and validation of a prognostic model, an independent statistical review would be beneficial especially with regard to the computed sample size and the maximum number of candidate predictors that may be included in the model.

**Reviewer**	**2**
**Name**	**Padhye, Bhavna**
**Affiliation**	**The Childrens Hospital at Westmead , sydney, australia, oncology**
**Date**	**02-Mar-2024**
**COI**	**Nil**

This is very important and interesting work and it will be useful to have a prognostic classification (risk stratification) for patients with Ewing sarcoma.

This model will be developed using data from EE2012 study, which means it is applicable when patients receive treatment as per EE2012 protocol, as prognostic (risk stratification) factors in the context of given protocol/treatment received.

Will this model be applicable/prognostic/risk predictable when patients are treated according to the next protocol INTER-EWING-1?

It will be useful if this is addressed specifically in the discussion that the model is in the context of the protocol and not general unless externally validated using different data set.

Also, there are multiple prognostic molecular markers described e.g. STAG -2,ct DNA, though this data is not routinely collected in many trials so far. It will be useful if this is discussed how this could be incorporated in the models.

**Reviewer**	**3**
**Name**	**Livinalli, Annemeri**
**Affiliation**	**UNICAMP**
**Date**	**09-Mar-2024**
**COI**	**None**

I recognize the importance of your future contribution, developing a prediction model for Ewing sarcoma treatment. Comparing with previous similar studies, you are proposing a more robut method.

I don't have any corrections. My only sugestions is to include in the section 1.3.6 the variables from the EE2012 trial.

**Reviewer**	**4**
**Name**	**Phillips, Bob**
**Affiliation**	**University of York, CRD**
**Date**	**22-May-2024**
**COI**	**None**

This is a clearly written protocol which seeks to develop a better prediction system using an individualised model than the current very coarse approach of localised / lung mets / other mets. The proposed method incorporates the 'best current guesses' for how to do this reliably and reproducibly, and will further the clinical objective and develop our understanding of which method might be the best.

Two queries arise which could be developed in the intro &/or incorporated into the protocol:

1. As there are no clear benefits to anything but VDC/IE in up-front treatment of ES, how will a more refined prediction actually affect clinical choices?

2. While not available at diagnosis, elements such as surgical excision vs. RT alone, and RT in oligometastatic disease (rx), along with tumour necrosis and PET-response (disease-response) have been suggested as important prognostic factors mid-treatment. Can the protocol suggest how these elements can be understood within the models as developed, and how this may affect utility should surgical approaches & technologies for RT / interventional radiology develop?

VERSION 1 - AUTHOR RESPONSE

Formatting Amendments (where applicable):

Reviewer: 1
Dr. Joseph Mathew, HealthCare Global Enterprises Ltd
Comments to the Author:
The study describes the protocol that would be followed in the development and internal validation of a prognostic prediction model to predict EFS in patients with Ewing sarcoma (ES) at presentation by the retrospective evaluation of the EE2012 data (the prognostic variables having been identified from a review of literature).

The manuscript is well written and once validated, the model would serve as a valuable resource for early prognostication in ES. I have the following comments to make:

1. The protocol for the EE2012 has been described in brief which is good. Please also consider mentioning the geographical distribution of patients included in the study since the same will have a bearing on generalizability and replicability of the model.

Firstly, thank you to Dr Joseph Mathew for taking the time to read this protocol and give constructive feedback.

The terminology "*European*" has now been added to the title which should hopefully provide transparency of the geographical distribution of patients. Similarly, text has now been added to the "Methods and Analysis: Participants" section of the manuscript to say "*from ten countries as described in the first results paper"* with a reference to the EE2012 results paper which details these countries and recruiting sites (reference 11).

2. With regard to the inclusion criteria, I assume that all patients with histology-proven ES and confirmed by molecular studies, regardless of organ of origin (skeletal vs extraskeletal) or metastatic status would be selected?

This is correct. The inclusion criteria (please see section "Methods and Analysis: Participants") states: "*any histologically and genetically confirmed Ewing sarcoma family of tumours (ESFT) of bone or soft tissue*". Similarly, bone vs soft tissue and metastatic status are both likely candidate predictors for the model, meaning we have data on these key patient attributes.

3. Please explain the rationale for including Ewing's like round cell neoplasms with conventional ESFTs in a prognostic model since it is now known that the biology and hence the presentation, response to therapy and aggressiveness of these tumors differ from that of classic ES.

These patients were included in the EE2012 study because they were treated the same as patients with classic Ewing sarcoma, meaning the study question was relevant for these patients. The reviewer rightly points out that these patients may have a different prognosis compared to patients with classic Ewing sarcoma, and therefore adjusting for this in the model might be necessary for more accurate and precise risk predictions. Moreover, this variable will be considered for inclusion in the set of candidate predictor parameters. Text has been added to the "Methods and Analysis: Candidate Predictors" to consider this:

An additional variable under consideration for inclusion in the set of candidate predictor parameters is whether the diagnosis was "Ewing's like" or not.

4. In the inclusion criteria, patients with "no prior treatment other than surgery" will be selected. Please clarify if this is a diagnostic biopsy that the authors are referring to or a surgical resection. In case of the latter, the surgical margin would represent an important prognosticator which cannot be overlooked.

It is possible for patients to enter the study if they have had a diagnostic biopsy and/or surgical resection. In total, 25 out of 640 patients had a surgical resection at diagnosis. The status of the surgical margins was reported for 20 of these patients. On the EE2012 trial's Surgery Case Report Form, the status of the patients' surgical margins are categorised by one of the following:

- R2 - macroscopic tumour remains post surgery
- R1a - Resection, even clear of active tumor cells, within post-chemotherapy fibrous reactive tissue (post chemotherapy necrotic tumor as well as reactive fibrosis) (Enneking marginal resection)

- R1b - Resection in close contact with tumor (less than 2mm without any normal anatomical structure/ Enneking marginal resection)
- R1c - Microscopical intralesionnal resection (viable tumor areas, tumor areas with coagulative necrosis (Enneking intralesional resection)
- R0 - Clear margins (free of viable, necrotic tumor and reactive tissue) 2mm or more of normal tissue in bone as well as in soft tissue or less than 2 mm if a normal anatomic structure (mostly fascia) can be identified (Enneking wide or radical resection)

Of the 20 patients where the status of surgical margins was reported following surgical resection at diagnosis, a small number of patients were categorised into each of the above. Furthermore, whilst we agree with the reviewer that this may be an important prognosticator, there is too little data to reliably inform the risk predictions. In addition, given the restraints of the sample size of this study (i.e., 11 candidate predictors can be included during variable selection), this variable is unlikely to be as informative as some of the other, more data rich, predictors. However, we will ensure that this is discussed in the results paper.

5. Although all predictor variables included in the model are routinely measured in practice and hence has functional value, a significant limitation of protocol studies deriving data from RCTs is that the data may not include newer, more potent prognosticators. Moreover, despite EFS being a composite survival end point, it is influenced by a number of modifiable treatment-related factors including systemic therapy and regimen, radiological and/or histological response, local treatment and margin status (if surgically addressed), all of which may not be accounted for if only baseline variables are considered to generate a prognostic model for EFS.

We completely agree with the reviewer's comment here. However, the purpose of this model will be to give clinicians and patients a risk prediction at diagnosis. We would not have a lot of information the reviewer is recommending at diagnosis. Our hope is to use this as a benchmark model which can be updated in the future to consider evolving information at later time points; as more information becomes available, patients' risk predictions can be updated to better suit the treatment decisions at hand.

6. Despite being a protocol study, I would expect a more detailed description of the prognosticators that would be included in the model, along with the rationale for selecting these variables and excluding others.

At the time the protocol was drafted and submitted in November 2023, the important discussions with clinicians and PPI around which of the potential predictors should be prioritised in the set of candidate predictor parameters had not yet been had. The narrative of prioritising certain potential predictors over others, within the sample size framework (i.e., 11 candidate predictors), was planned to go into the results. We understand the reviewer's perspective, and rather than discussing the data in detail within the manuscript, we have opted to add more text to explain exactly how the set of candidate predictors will be chosen and which of the potential predictors are collected at diagnosis in EE2012. Please see the additional text in section "Methods: Candidate Predictors" to clarify this, and again here:

these include metastatic site, number of metastases, tumour volume, location of primary tumour, age and sex. Additional variables under consideration for inclusion in the set of candidate predictor parameters are whether the diagnosis was "Ewing's like" or not. Moreover, the set of

candidate predictors will be chosen based on clinician and patient opinion, and within the limitations of the EE2012 dataset.

7. Also, I'm uncertain as to why the authors have included both tumor size and volume as candidate predictors, since the latter may be considered a function of size (unless indicative of overall tumor burden) and hence a more comprehensive parameter when compared to the largest dimension of a tumor.

Both tumour size and tumour volume were being quoted as potential candidate predictors based on systematic reviews looking at prognostic factors and previous prognostic model development studies in newly diagnosed Ewing sarcoma patients. These potential candidate predictors will be limited to the actual set of candidate predictor parameters as part of the results based on their frequency in the literature, conversations with clinicians and PPI, and limitations of the EE2012 trial data. We appreciate the current wording may not be entirely clear on this point. Please see the revised text within the manuscript in section "Methods and Analysis: Candidate Predictors" and below, where the bold text highlights in particular aims to clarify this point:

Several potential predictors of EFS in Ewing sarcoma have previously been identified in systematic reviews [13, 14]. Similarly, previous prognostic modelling studies in newly diagnosed Ewing sarcoma have been conducted [3-6]. From those, the potential predictors that may have prognostic ability and are measured at diagnosis includes metastatic site, number of metastases, tumour size, tumour volume, location of primary tumour, age, serum LDH levels, sex and race. ***As part of the proposed research, these potential predictors will be limited to a set of 11 candidate predictor parameters.*** *It is important that the predictors considered are endorsed by clinical experts in Ewing sarcoma. In addition, only variables that were routinely collected at diagnosis as part of the EE2012 trial will be included in the set of candidate predictors; these include randomised treatment, metastatic site, number of metastases, tumour volume, location of primary tumour, age and sex. An additional variable under consideration for inclusion in the set of candidate predictor parameters is whether the diagnosis was "Ewing's like" or not.* ***Moreover, the set of candidate predictors will be chosen based on clinician and patient opinion, within the limitations of the EE2012 dataset.***

8. Also, the EE2012 being a European trial, how "variable" a candidate predictor would race be?

As explained above, this is not a candidate predictor, but a potential predictor that has previously been discussed in systematic reviews. Race was not collected as part of the EE2012 trial and therefore it would not be possible to include race as a candidate predictor. In the future however we hope to consider race in the model to better understand the fairness of the model, i.e., ensure the risk predictions from the model can be generalised to all groups of patients, regardless of race.

9. How would the authors assess for risk of bias?
At the end of the study, the advantages and limitations of the study will be discussed in the context of PROBAST. Text has been added to the section "Methods and Analysis: Statistical Analysis Plan – Reporting" to cover this point:

In addition, the advantages and disadvantages of this study will be discussed in the context of the PROBAST tool [32].

Minor comments and corrections:
1. The title of the study reads as "Developing and internally validating a *clinical* prediction model...". For clarity, I would suggest modifying this to a "*prognostic* prediction model" as clinical prediction models could either imply diagnostic or prognostic prediction models.

Development and internal validation of prognostic models for event-free survival in newly diagnosed Ewing sarcoma patients based on routinely collected clinical characteristics from the European randomised controlled trial, EE2012: Protocol

This terminology (i.e., prognostic model instead of clinical prediction model) has been updated throughout the manuscript.

2. Kindly recheck the references especially the sources that have been quoted from the net.
The references have now been checked and updated where appropriate.

Reviewer: 2
Dr. Bhavna Padhye, The Childrens Hospital at Westmead , sydney, australia
Comments to the Author:
This is very important and interesting work and it will be useful to have a prognostic classification (risk stratification) for patients with Ewing sarcoma.

This model will be developed using data from EE2012 study, which means it is applicable when patients receive treatment as per EE2012 protocol, as prognostic (risk stratification) factors in the context of given protocol/treatment received.

Will this model be applicable/prognostic/risk predictable when patients are treated according to the next protocol INTER-EWING-1?

Thank you to Dr Bhavna Padhye for taking the time to read this manuscript and offering constructive feedback.

This model will be applicable to the INTER-EWING-1 study, and in fact one of the rationales for developing this model is to potentially provide better risk stratification for patients entering the study, and in particular the frontline chemotherapy randomisation (i.e., standard of care vs standard of care with Regorafenib). Treatment will be forced into the prognostic model as a covariate and therefore patients baseline risk score will be adjusted for the frontline chemotherapy regimen they receive (i.e., VDC/IE). The model will need to be updated as and when new treatments are identified as having a treatment effect.

It will be useful if this is addressed specifically in the discussion that the model is in the context of the protocol and not general unless externally validated using different data set.

This comment is very valid and a sentence has now been added to the “Discussion” section to demonstrate the direct impact this could have on INTER-EWING-1. Please also see below:

If a model demonstrates good predictive performance when validated, it could be implemented into the next large international randomised controlled trial in newly diagnosed Ewing sarcoma, INTER-EWING-1, to aid decisions around which randomisations patients could enter based on their risk prediction, e.g., randomising the high-risk patients to standard of care vs standard of care with a novel therapy.

Also, there are multiple prognostic molecular markers described e.g. STAG -2,ct DNA, though this data is not routinely collected in many trials so far. It will be useful if this is discussed how this could be incorporated in the models.

The evidence of the prognostic effect of the specific biomarkers mentioned here are variable and the sample size of these biological studies a major limitation. However, once a benchmark model is developed with clinical variables, as proposed in this protocol, the resulting model can be updated to incorporate prognostic biomarkers in the future. A sentence within the "Discussion" section touches on the potential of this future research:

In addition, the resulting models have the potential to be updated as additional prognostic factors derived from translational research (i.e., biomarkers) arise, which may give more accurate prediction.

Reviewer: 3
Dr. Annemeri Livinalli, UNICAMP
Comments to the Author:
I recognize the importance of your future contribution, developing a prediction model for Ewing sarcoma treatment. Comparing with previous similar studies, you are proposing a more robust method.
I don't have any corrections. My only suggestion is to include in the section 1.3.6 the variables from the EE2012 trial.

Thank you to Dr Annemeri Livinalli for taking the time to review this protocol. Based on your comment, the following text has now been added to the "Methods and Analysis: Candidate Predictors" section:

these include metastatic site, number of metastases, tumour volume, location of primary tumour, age and sex.

Reviewer: 4
Dr. Bob Phillips, University of York
Comments to the Author:
This is a clearly written protocol which seeks to develop a better prediction system using an individualised model than the current very coarse approach of localised / lung mets / other mets. The proposed method incorporates the 'best current guesses' for how to do this reliably and reproducibly, and will further the clinical objective and develop our understanding of which method might be the best.

Two queries arise which could be developed in the intro &/or incorporated into the protocol:

1. As there are no clear benefits to anything but VDC/IE in up-front treatment of ES, how will a more refined prediction actually affect clinical choices?

Thank you to Dr Bob Phillips for taking the time to review this protocol and offering useful feedback.

This comment is similar to one previously addressed from another reviewer and hopefully has been clarified. The next large international randomised controlled trial in newly diagnosed Ewing sarcoma, INTER-EWING-1, has multiple randomisations patients can enter. Currently patients' disease type at diagnosis is the only prognostic factor being used to decide which randomisation patients could enter. For example, patients with metastatic disease are being offered the opportunity to enter a randomisation between standard of care and standard of care with Regorafenib for their frontline chemotherapy. However, some metastatic patients respond well to treatment and might not truly be "high-risk", whereas some localised disease patients do very poorly and may benefit from the novel therapy. The prognostic model developed from this work may help to better identify the high and low risk patients to enter certain randomisations. Additional text has now been added to the discussion to clarify this point:

If a model demonstrates good predictive performance when validated, it could be implemented into the next large international randomised controlled trial in newly diagnosed Ewing sarcoma, INTER-EWING-1, to aid decisions around which randomisations patients could enter based on their risk prediction, e.g., randomising the high-risk patients to standard of care vs standard of care with a novel therapy.

2. While not available at diagnosis, elements such as surgical excision vs. RT alone, and RT in oligometastatic disease (rx), along with tumour necrosis and PET-response (disease-response) have been suggested as important prognostic factors mid-treatment. Can the protocol suggest how these elements can be understood within the models as developed, and how this may affect utility should surgical approaches & technologies for RT / interventional radiology develop?

We completely agree with the reviewers comment here. However, the purpose of this model will be to give clinicians and patients a risk prediction at diagnosis. We would not have a lot of information the reviewer is recommending at diagnosis. Our hope is to use this as a benchmark model which can be updated in the future to consider evolving information at later time points; as more information becomes available, patients' risk predictions can be updated to better suit the treatment decisions at hand.

VERSION 2 - REVIEW

**Reviewer**	**1**
**Name**	**Mathew, Joseph**
**Affiliation**	**HealthCare Global Enterprises Ltd**
**Date**	**22-Nov-2024**

COI

The authors have satisfactorily addressed the concerns expressed by the reviewers in the revised version of the manuscript.

**Reviewer**	**4**
**Name**	**Phillips, Bob**
**Affiliation**	**University of York, CRD**
**Date**	**11-Nov-2024**
**COI**	

This revision has clarified the minor issues which were present in the original report
